# Regulatory Mismatch and Transboundary Governance of Migratory Game Birds: A Comparison of Poland and Germany

**DOI:** 10.1007/s00267-026-02584-0

**Published:** 2026-09-16

**Authors:** Kinga Piórkowska, Wiktor Strączyński, Łukasz Jankowiak, Michał Polakowski

**Affiliations:** 1 Department of Ecology and Anthropology, Institute of Biology, University of Szczecin, Szczecin, Poland; 2 Doctoral School of the University of Szczecin, Szczecin, Poland; 3 Department of Law and Administration, University of Szczecin, Szczecin, Poland

**Keywords:** Adaptive harvest management, Hunting legislation, Game birds

## Abstract

Hunting governance in Europe blends territorial and licensing traditions, producing hybrid regulatory regimes. We compared Poland’s centralised hunting act with Germany’s federal state-level regulations to examine how governance architecture was associated with season design, legal game status, and reported harvest for shared migratory game birds. We combined comparative legal analysis with national bag statistics from 12 hunting seasons (2012/2013–2023/2024) and available abundance estimates for wood pigeon, Eurasian woodcock, and three *Anser* geese taxa. Germany had higher indicative harvest-to-abundance ratios for wood pigeon (≈6–11%) and woodcock (≈13–20%) than Poland (≈0.5–0.6% and <1%), whereas geese had higher ratios in Poland (≈22–27%) than Germany (≈13–18%) when interpreted against wintering or migration-period reference populations. Findings are descriptive and should not be interpreted as demographic harvest mortality. They highlight a transboundary governance mismatch and support harmonised harvest reporting, explicit reporting of population-reference phases, and cautious pilot testing of adaptive management where monitoring and institutional capacity are sufficient.

## Introduction

Hunting in Europe is viewed with ambivalence. Harvesting can influence population status and, in turn, ecological balance and biodiversity conservation (Daniłowicz, 2020), while also sparking controversy over killing methods and traditions (Michalska, 2020). Because hunting removes individuals, it may contribute to unfavourable outcomes in some taxa, such as the broadly declining common pochard *Aythya ferina* (BirdLife International, 2025), and effects may be especially variable in habitat-specialist species or species with restricted ranges, such as hazel grouse (*Tetrastes bonasia)* (Matysek, 2018). Originally a subsistence activity, hunting became increasingly regulated and modernised, incorporating elements of active and passive environmental protection (Gawin et al. 2015), and today also serves recreational and social functions (Maćkowiak and Budych-Tomkowiak, 2012).

Europe has developed two main hunting models. A territorial, district-based model ties hunting rights to legally delimited hunting districts or ranges, usually exercised by a leaseholder or management body responsible for wildlife management within that area. A licensing model, by contrast, links hunting access primarily to individual permits, registries or licences, usually combined with landowner permission and administrative oversight. In practice, contemporary hunting systems rarely correspond fully to either ideal type. Poland and Germany both combine territorial and licensing elements, but they do so in different ways. Poland represents a strongly centralised district-based system, in which hunting is organised through leased hunting districts and embedded within the Polish Hunting Association structure. Germany also retains district-based hunting rights but combines them with stronger state-level regulatory competence, more decentralised leaseholding arrangements and greater regional discretion in setting or modifying hunting rules. Such hybrid systems may combine effective population management with wider participation, thereby promoting conservation and public acceptance (Dzięciołowski and Mattioli, 2015).

Despite extensive descriptions of national hunting systems in Europe, we still lack comparative, evidence-linked assessments of how governance architecture (centralised vs. federal regulatory competence) translates into measurable regulatory outcomes, such as season timing, legal game lists and realized harvest for shared transboundary populations. This gap matters because the EU Birds Directive allows hunting only under conditions compatible with conservation objectives (Directive 2009/147/EC), and because differences in season timing and harvest intensity across borders may influence stakeholder conflicts among agriculture, hunting and nature conservation, as well as complicate flyway-scale management. At the same time, regulatory differences are not necessarily undesirable. Spatially or temporally differentiated rules may allow authorities to account for regional variation in migration phenology, local abundance, agricultural damage and conservation priorities. The governance challenge is therefore not to eliminate all regulatory variation, but to distinguish adaptive regional differentiation from uncoordinated fragmentation affecting the same mobile populations.

To ground this comparison in shared, transboundary wildlife, we focus on five game-bird taxa occurring in both Poland and Germany: the common wood pigeon (*Columba palumbus)*, the Eurasian woodcock (*Scolopax rusticola)*, and three *Anser* goose taxa: greylag goose (*Anser anser*), the bean goose complex, and greater white-fronted goose (*Anser albifrons*). We selected these taxa because they (i) are regulated and harvested in both hunting systems (at least during part of the study period), (ii) span contrasting ecological guilds and management contexts (forest-associated woodcock, crop-associated geese, and an agricultural generalist pigeon), (iii) represent migratory populations for which cross-border coordination is most relevant under EU conservation obligations, and (iv) have publicly available abundance and bag statistics enabling indicative harvest-to-abundance comparisons.

Neighbouring Poland and Germany share rich hunting traditions, yet their legal frameworks differ markedly and therefore create different regulatory opportunity spaces for harvest. Because legal rules determine which taxa can be harvested and when, and harvest statistics show what is taken in practice, we combine comparative legal analysis with quantitative harvest and abundance data to compare governance architectures in terms of season design, legal opportunity space, and reported harvest patterns. Specifically, we (i) compare the statutory game list and the timing/length of open seasons, (ii) quantify cross-country differences and temporal trends in reported harvest for the focal taxa, and (iii) relate harvest to available reference population estimates to provide indicative harvest-to-abundance comparisons. We expected the German federal framework to create greater within-country heterogeneity and more scope for local adjustment of season design, whereas Poland’s centralised framework was expected to yield more uniform rules. We did not directly assess implementation quality, enforcement intensity, or population-level effects of harvest. We frame these expectations as comparative research questions about regulatory opportunity space and reported harvest patterns, rather than hypotheses about causal effects of governance on population outcomes.

## Data and Material Resources

### Study Area

**Fig. 1:**
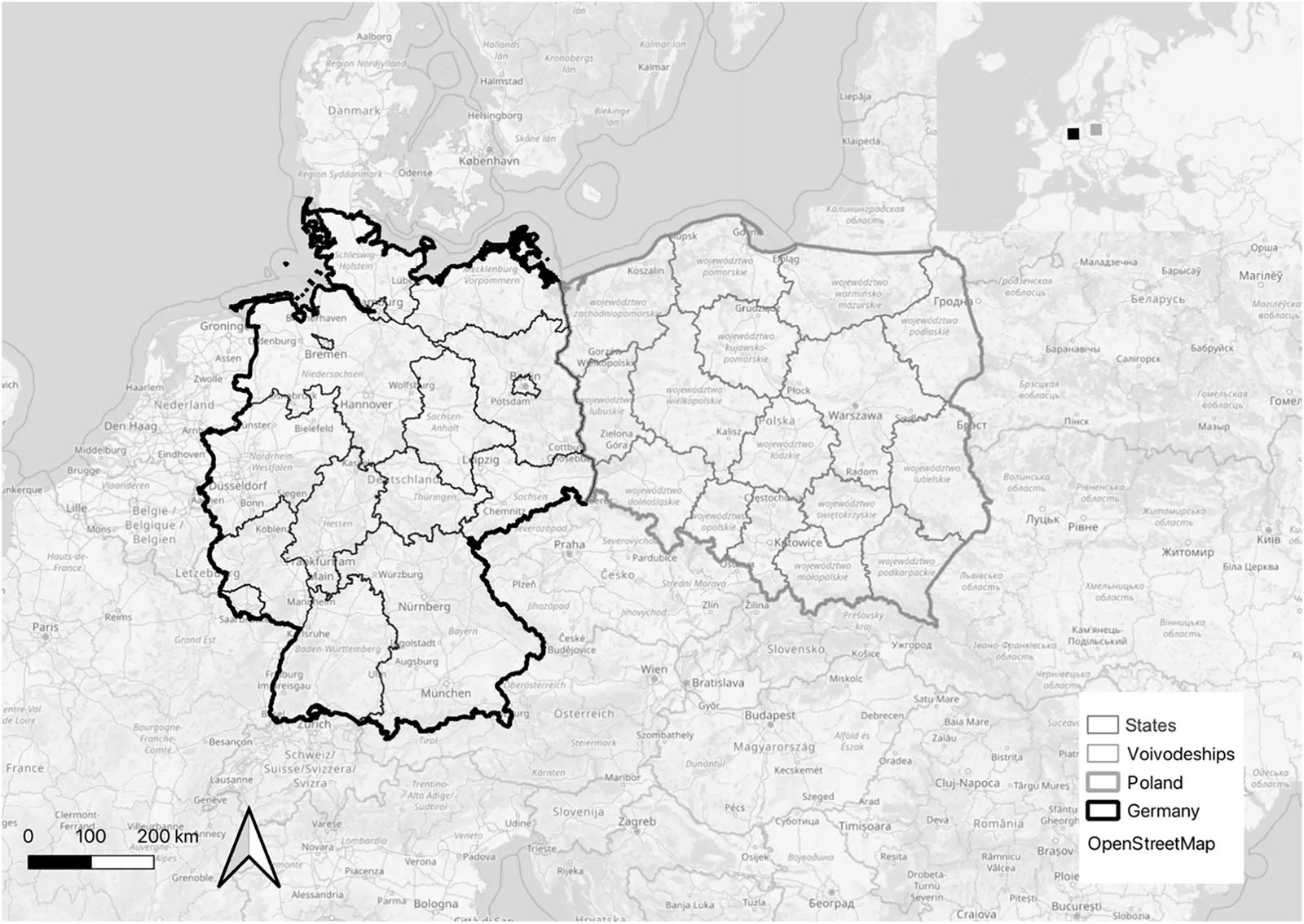
Location of the study area

The study area covered two neighbouring countries, i.e., Poland and Germany (Fig. 1). Poland is divided into 16 voivodeships, each subordinate to the central government, whereas Germany is made up of 16 states (Länder) within a federal system that retains broad legislative and executive powers. The two countries share a total length of 467 km land border.

Poland covers 312,685 km² of land area, forest covers 29.44% of that area. Agricultural land makes up about 59.5%, and surface waters-lakes, rivers, and wetlands-occupy roughly 2.2% (Wojciechowska et al. 2024). Among European Union member states, Poland ranks fourth in terrestrial protected-area coverage: conservation categories encompass 32.2% of the country (2022 data). Germany, with a total area of 357,683 km², is about 29.9% forested (Statistisches Bundesamt, 2023). Agriculture uses 50.3% of Germany’s land, while surface waters-lakes, rivers, and reservoirs-account for around 2.3% (Statistisches Bundesamt, 2023). Germany ranks seventh in the EU for terrestrial protected-area share, area-based measures cover roughly 31.5% of its territory (Bach and Larondelle, 2023; Wojciechowska et al. 2024). The ecological and geographic similarity of these neighbouring countries, along with their shared border, provides a strong basis for comparing hunting practices.

### Study Species, Harvest Data and Analyses

#### Study Species

The greater white-fronted goose breeds on the Eurasian tundra. In Poland and Germany, it appears only during migration and winter and is one of the most numerous wintering geese in both countries. Birds leave for wintering grounds from September to November and return from February to May, breeding occurs from May to July (e.g., Kölzsch et al. 2016; Kölzsch et al. 2020).

In the present study, the term “bean goose” is used as a collective reporting category that includes both Tundra Bean Goose (*Anser serrirostris*) and Taiga Bean Goose (*Anser fabalis*). This approach reflects the structure of national legal and harvest-reporting categories, which do not consistently distinguish between these taxa. In ecological terms, however, the Tundra Bean Goose clearly predominates in the study area, particularly among wintering birds in Germany and western Poland, and its wintering population is increasing (Marjakangas et al. 2015). Autumn migration usually occurs from September to November, with return migration in March and April; the breeding season lasts from May to July (Heinicke, 2010; Nilsson, 2011; Ottenburghs et al. 2020; Fox et al., 2021; Piironen et al. 2021). Although the two taxa differ in morphology, range and population trends (Ottenburghs et al. 2023), Polish law has not yet adopted this taxonomic split. Moreover, the western taiga population of *Anser fabalis* is largely panmictic across Finland, but elevated inbreeding coefficients, high relatedness in some birds and introgression from the pink-footed goose (*Anser brachyrhynchus*) signal emerging genetic risks, underscoring the need to manage the entire flyway as an integrated unit and to monitor inbreeding and hybridisation (Honka et al. 2022).

The greylag goose is widespread across Europe, Asia, and North Africa and nests in wetlands, lakes, and reedbeds in Poland and Germany. Northern birds migrate to western Europe and the Mediterranean from September to November and return from February to April, but an increasing number of individuals are resident and overwinter in both countries. Breeding runs from March to June (Anderson et al. 2001; Pistorius et al. 2006; Voslamber et al. 2010; Nilsson, 2017; Nilsson and Kampe-Persson, 2018; Bacon et al. 2019; Guillemain et al. 2019). In national bag statistics, geese were reported as an aggregated category (*Anser spp*.); therefore results for geese are presented at the genus-aggregate level.

The common wood pigeon ranges across the western Palearctic and is partially migratory: northern and eastern populations move south from September to November and return in March and April, while many western birds remain sedentary, including some in Poland and Germany. Breeding in the study area spans April to September, and pairs may produce multiple broods per season (Bankovics, 2001; Cohou et al. 2006; Hobson et al. 2009; Butkauskas et al. 2019; BirdLife International, 2025).

Similarly to the common wood pigeon, the Eurasian woodcock has a comparable Palearctic range and migratory pattern. Northern and eastern birds move southward between September and November and return in March and April, whereas many western birds overwinter locally. A secretive species, it breeds in April and May in the study area (Cramp and Simmons, 1983; Hoodless, 1995; Tavecchia et al. 2002; Peron et al. 2011; Peron et al. 2012; Arizaga et al. 2014; Guzmán et al. 2017; Prieto et al. 2019).

The study considered three types of information for each focal taxon: (i) population status, based on monitoring programs and reports; (ii) hunting seasons and (iii) reported harvest size, expressed as the number of individuals taken during the 2012/2013-2023/2024 hunting seasons in both countries.

#### Harvest Data, Harmonisation and Statistical Analysis

All analyses refer to the period 2012/2013–2023/2024, during which Eurasian woodcock was still listed as a game species in Poland. Subsequent legal changes removing woodcock and other bird species from the national game list from 2 January 2026 onward are therefore outside the temporal scope of our dataset.

Annual harvest data were obtained from official national hunting-bag statistics: for Poland, from the Research Station of the Polish Hunting Association in Czempiń, published in the series “*Sytuacja zwierząt łownych w Polsce - wyniki monitoringu*” and “*Zestawienie danych sprawozdawczości łowieckiej”* (Stacja Badawcza PZŁ Czempiń, n.d.), and for Germany, from the hunting-statistics portal of the Deutscher Jagdverband (DJV), *Jagdstatistik für einzelne Wildarten*, including the national annual hunting-bag overview *Gesamtübersicht - Jahresjagdstrecke Bundesrepublik Deutschland* and species-specific annual hunting-bag summaries (Deutscher Jagdverband, n.d.). National harvest statistics were not fully taxonomically equivalent between Poland and Germany because German reporting aggregates multiple species into “basket” categories, e.g., *Wildtauben* and *Wildgänse*, whereas Polish reported totals correspond directly to the analysed taxa. To ensure cross-country comparability, we analysed two harvest series: (i) the raw basket totals as reported nationally and (ii) a harmonised common-species series representing taxa shared between both hunting systems, which was used for all cross-country comparisons. For Poland, raw and harmonised values were identical and no correction was applied. Polish national hunting-bag data were used exactly as reported in the official summaries. For Germany, harmonised series were derived from DJV national basket totals using state-level hunting reports that provided species-level breakdowns, particularly reports from North Rhine-Westphalia and Lower Saxony (Ministerium für Landwirtschaft und Verbraucherschutz des Landes Nordrhein-Westfalen, 2024; Gräber et al. 2023; Gräber et al. 2025). For pigeons, *Wildtauben* totals were converted to an estimated wood pigeon series by applying the proportional share of *Ringeltaube* (central *p* = 0.995; sensitivity: 0.992-0.9993 based on the observed regional range). For geese, *Wildgänse* totals were reduced by the estimated contribution of non-native geese, notably Egyptian goose (*Alopochen aegyptiaca*) and Canada goose (*Branta canadensis*), using state-level species data. Main results are based on harmonised series- raw basket totals are reported for transparency in Supplementary Material S1.

For each taxon and country, we calculated the geometric mean annual harvest across the study period and expressed cross-country differences as a geometric mean DE/PL ratio with 95% confidence intervals (CI), obtained from log-transformed annual ratios and tested against a ratio of 1. Temporal trends in harvest were estimated with log-linear models fitted separately for each taxon and country: log(bag) ~ season (season coded as a numeric covariate). Slopes were reported as percentage change per season with 95% CI and p-values from standard model inference. To test whether temporal trends differed jointly by country and taxon, we fitted a global interaction model: log(bag) ~ country × taxon × season. For illustrative comparisons, we calculated annual harvest-to-abundance ratios as reported bag divided by the most comparable publicly available population estimate. Where population sizes were given as ranges, we reported ratio intervals based on minimum and maximum estimates. Population estimates were assigned to the phenological phase to which they refer: breeding estimates for common wood pigeon and Eurasian woodcock, and wintering or migration-period estimates for geese where available; the phenological phase and source of each population estimate are provided in Supplementary Material S3. These ratios should therefore not be interpreted as demographic harvest mortality or as direct pressure on local breeding populations. This is particularly important for autumn and winter harvest, which may include migrants originating from breeding strongholds outside Poland or Germany. The ratios are used only as indicative measures of the scale of reported harvest relative to available reference populations. Because the series is short, we did not attempt to model more complex temporal dependence; therefore results should be interpreted as descriptive trend estimates. All analyses were conducted in R (version 4.2.3; R Core Team 2023).

Our design compares formal regulatory architecture and reported harvest statistics. It does not directly measure enforcement intensity, local administrative discretion, hunter behaviour, compliance, or how legal provisions are implemented in practice. Accordingly, the study should be read as a comparison of governance design and reported harvest patterns, not as a full evaluation of implementation effectiveness.

### Game-management Framework and Institutional Context

To characterise hunting management and institutional context in both countries, we conducted a comparative analysis of the key statutes and regulations governing hunting, together with the main supplementary legal acts (S2). Because harmonised species-specific hunting-effort data, such as hunter-days, hunting hours or the number of hunting trips directed at each focal taxon, were not available for both countries, this section focuses on formal governance design and the institutional context of hunting activity rather than on effort-corrected harvest estimates. The purpose of this analysis was to characterise the legal opportunity space for management, rather than to directly observe implementation practices in the field. A four-step comparative doctrinal analysis was applied: (i) selection of sources and mapping of relationships among the acts; (ii) coding criteria, including list of game species, length and structure of seasons, hierarchy of management authorities, monitoring obligations, derogation mechanisms and sanctions; (iii) content analysis; (iv) synthesis and validation.

## Results

### Game species

The common wood pigeon and the greylag goose increased in both Poland and Germany, consistent with broader European trends. Available national estimates for common wood pigeon refer to breeding populations, exceeding 1 million breeding pairs in Poland and 3 million in Germany. For Eurasian woodcock, available national estimates also refer to breeding populations: Poland holds an estimated 20,000–100,000 breeding pairs, whereas Germany supports 20,000-39,000 breeding pairs. In contrast, goose estimates used for interpretation refer mainly to wintering or migration-period abundance rather than breeding populations. Wintering and migration-period estimates, including International Waterbird Census (IWC) data, indicate approximately 92,000 migrant greylag geese in Poland and 244,000 in Germany, 120,000–130,000 migrant greater white-fronted geese in Poland and 300,000-320,000 in Germany, and 7000-23,000 wintering bean geese in Poland and 11,500 in Germany. The phenological phase and source of each population estimate are provided in Supplementary Material S3. Key regulatory differences concern season timing and internal heterogeneity. In Poland, a single nationwide regulation sets seasons at 15 Aug-30 Nov for wood pigeon and 1 Sep-21 Dec for woodcock, and a nationwide goose season of 1 Sep-21 Dec (extended to 15 Jan only in four western voivodeships). In Germany, federal baseline seasons open later (wood pigeon 1 Nov-31 Jan; woodcock 16 Oct-15 Jan) and state-level regulations may further refine dates; for geese, Germany applies a shorter, two-part window (1 Aug-31 Aug and 1 Nov-15 Jan) that individual states may adjust (S3). According to the phenological information summarised in S3, the hunting seasons analysed here overlap mainly with post-breeding dispersal, autumn migration, staging or wintering periods rather than with the core breeding period, although the relative importance of these phases differs among taxa and countries. To avoid interpreting harvest-to-abundance ratios as direct demographic pressure on local breeding populations, each reference estimate was assigned to its phenological phase and interpreted with the limitations summarised in Table 1. Harvest-to-abundance ratios are therefore interpreted as descriptive indicators of reported harvest relative to available reference populations, not as direct estimates of demographic harvest mortality.

**Table 1:** Phenological context and interpretation limits of the reference population estimates used in harvest-to-abundance comparisons

Taxon	Reference estimate used	Phenological phase of reference estimate	Main limitation for interpretation
Common wood pigeon	National breeding-population estimate	Breeding population	Reported harvest may include migrants or post-breeding dispersers, not only local breeders
Eurasian woodcock	National breeding-population estimate	Breeding population	Autumn/winter harvest may include non-local birds from other breeding areas
Greylag goose	IWC migration-period counts used for harvest-to-abundance comparison; breeding and wintering estimates also reported in S3	Wintering/migration period	Birds recorded or harvested may originate from several breeding areas
Greater white-fronted goose	IWC migration-period counts used for harvest-to-abundance comparison; wintering estimates also reported in S3	Wintering/migration period	No regular local breeding population; interpretation refers to migratory/wintering birds
Bean goose	National wintering-population estimate	Wintering population	Taxonomic and flyway uncertainty; “bean goose” is treated as a collective reporting category

**Fig. 2:**
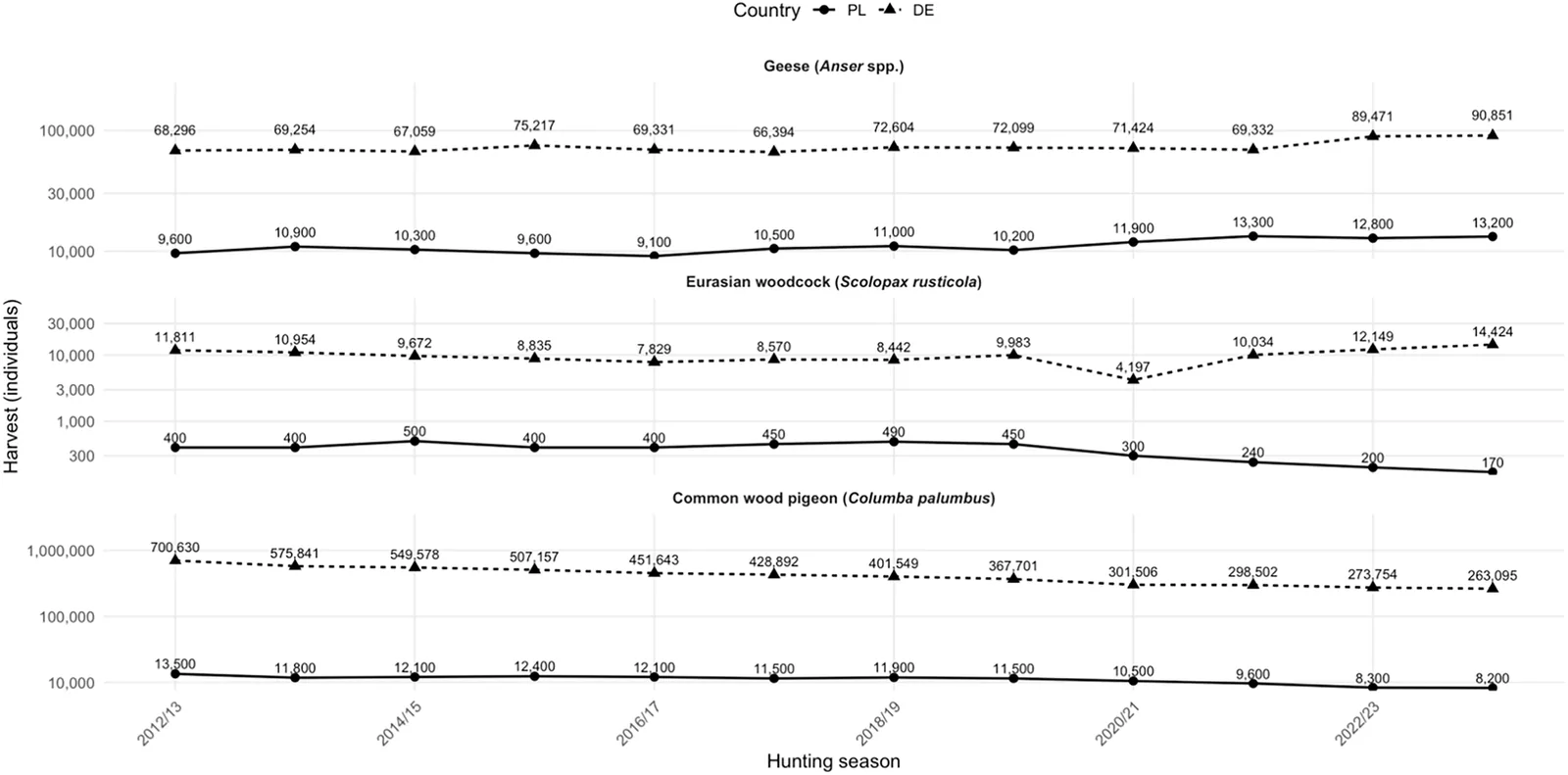
Annual harvest of geese (*Anser spp*.), Eurasian woodcock (*Scolopax rusticola*) and common wood pigeon (*Columba palumbus*) in Poland (PL) and Germany (DE), 2012/2013-2023/2024

Across twelve hunting seasons (2012/2013–2023/2024), reported harvest (bag) levels were consistently higher in Germany than in Poland for all analysed taxa (Fig. 2). Averaged over the study period, Germany harvested 73,444 geese (*Anser spp*.) per season (66,394–90,851) versus 11,033 in Poland (9100–13,300), corresponding to a geometric mean DE/PL ratio of 6.67 (95% CI: 6.22–7.15; *p* < 0.001). For Eurasian woodcock, Germany reported 9742 birds per season (4197–14,424) compared with 367 in Poland (170–500), i.e. a 26.97-fold higher harvest on average (95% CI: 19.13–38.04; *p* < 0.001). The strongest absolute contrast was observed for common wood pigeon, with German harvest averaging 426,654 birds per season (263,095-700,630) versus 11,117 in Poland (8200-13,500), yielding a 37.04-fold difference (95% CI: 32.81–41.82; *p* < 0.001).

Interannual trajectories were taxon-specific and differed between countries (Fig. 2). Log-linear trend models indicated that goose harvest increased over time in both countries, by +2.91% per season in Poland (*p* = 0.0018) and +1.99% per season in Germany (*p* = 0.0149), with maxima in 2021/2022 in Poland (13,300) and 2023/2024 in Germany (90,851). In contrast, woodcock harvest declined in Poland (−7.34% per season, *p* = 0.0042), reaching the series minimum in 2023/2024 (170), whereas Germany showed no directional trend (*p* = 0.962) but substantial year-to-year variability (minimum in 2020/2021, 4197; maximum in 2023/2024, 14,424). Wood pigeon harvest decreased in both countries, with a markedly steeper decline in Germany (−8.38% per season, *p* < 0.001) than in Poland (−3.85% per season, *p* < 0.001). Accordingly, the DE/PL harvest ratio for wood pigeon decreased significantly over time (approx. −4.71% per season, *p* < 0.001). Consistent with these patterns, a global model detected a significant time × country × taxon interaction (*p* = 0.0002), indicating that temporal changes in harvest magnitude depended jointly on country and taxon.

Although our formal comparisons were based on national-level harvest series, available state-level information indicates substantial within-Germany variation in management context. This variation is reflected primarily in state-level modifications of hunting seasons and damage-related derogations. For example, Brandenburg allows additional goose-hunting periods on fields with documented crop damage, Sachsen-Anhalt authorises hunting when at least 50 geese are feeding on crops, Schleswig-Holstein extends the goose season to 31 January while prohibiting harvest within Natura 2000 sites, and Hesse has maintained a long-standing moratorium on Eurasian woodcock hunting. State-level hunting reports were also used where species-level breakdowns were needed to harmonise German basket categories, such as *Wildtauben* and *Wildgänse*; however, because taxonomic resolution and temporal availability differed among federal states, these data were used descriptively and for harmonisation rather than for formal state-level trend analysis.

### Game Management Framework and Institutional Context (S2)

As direct species-specific hunting-effort metrics were unavailable, we did not calculate effort-corrected harvest indices. Instead, national bag statistics are interpreted in relation to the legal and institutional context of hunting activity. As general context, the Polish Hunting Association reports over 130,000 hunters and 2800 hunting clubs, whereas the Deutscher Jagdverband (DJV) reports 467,682 licensed hunters in Germany based on November 2025 data (Polski Związek Łowiecki 2026; Deutscher Jagdverband 2026). These figures describe the potential hunting community rather than effort directed at the focal taxa.

In Poland, leaseholders manage hunting within district boundaries under annual harvest plans and ten-year game-management plans. For birds, planning requirements are less stringent than for ungulates: migratory game birds are explicitly exempt from the statutory pre-season inventory (Art. 8a sec. 2). Instead, leaseholders record a planned take for each bird species and define a permissible range equal to 85–115% of that value (Art. 8a sec. 6 pt 4 let. h-i), which in practice is usually anchored in the previous season’s reported bag. Thus, for migratory game birds, annual planning is largely path-dependent, rather than tied to explicit abundance targets. This interpretation is consistent with recent evidence from Poland showing substantial discrepancies between planned and realised waterbird take, with realised values often far below planned ceilings (Piórkowska et al. 2026). This highlights the need to better align harvest planning with monitoring data and actual hunting activity.

Ultimate authority in hunting matters rests with the minister of the environment, while day-to-day administration falls to the voivodes (Art. 6-7). After consultation, the minister sets the national game list and hunting seasons by regulation (Art. 5). Free-living animals belong to the State Treasury; ownership passes to the leaseholder only after lawful harvest inside the district (§ 2, 15). Hunting is limited to members of the Polish Hunting Association with the leaseholder’s consent. Each district keeps an electronic attendance log that hunters must complete before hunting; these data are publicly available on request (Art. 42b secs 1-1 d). Statutory compensation covers only damage caused by large mammals, whereas losses from rapidly increasing wintering goose flocks fall outside the mandatory scheme and are dealt with, if at all, through separate arrangements (Polakowski et al. 2023). These institutional features are particularly relevant for migratory game birds because they shape how harvest plans are set and how conflicts over crop damage can be addressed.

In Germany, leaseholders or landowners manage hunting ranges under annual harvest plans within a federal framework. The hunting year runs from 1 April to 31 March, and electronic harvest plans must be filed by 15 March. Many federal states also require multi-year game-management plans drawn up within cooperatives that group neighbouring ranges sharing similar habitat. Free-ranging game is not owned until the moment of harvest (§ 1 para. 1; § 3), and hunting rights are tied to hunting ranges, whether private or communal, rather than to individual hunters. The Federal Hunting Act states that the primary objective of hunting is to prevent agricultural, forestry and fishery losses by holding game populations at non-damaging levels (§ 1 para. 2), which strongly influences the management of herbivorous birds such as geese and, to a lesser extent, wood pigeons.

Hunting permits are issued by the authority for the hunter’s place of residence and require passing a state hunting examination; youth licences allow hunting under supervision. Membership in the Deutscher Jagdverband is not mandatory. Statutory crop-damage compensation under § 29 of the Federal Hunting Act covers cloven-hoofed game, wild rabbits and pheasants, but not geese or other birds. Where goose damage is addressed, it is typically through separate state-level programmes. For example, Lower Saxony’s “goose grazing peaks” scheme and, since May 2025, a similar fund in Schleswig-Holstein reimburse documented goose damage up to a defined ceiling per farm, financed from nature-conservation funds under § 68 of the Federal Nature Conservation Act. Such arrangements provide an important background to the harvest patterns we observe for migratory geese, since they may influence both tolerance thresholds and incentives for lethal control. Key structural differences between the Polish and German systems are summarised in Table 2. To address their relevance for the present study, we distinguish provisions directly affecting migratory game-bird management, such as game-species lists, hunting seasons, planning rules, derogation mechanisms and damage-related provisions, from broader institutional features that shape governance capacity and implementation context, such as lease structure, membership rules and allocation of hunting rights.

**Table 2:** Key differences in hunting regulation between Poland and Germany, distinguishing provisions directly relevant to migratory game-bird management from broader institutional features shaping governance context

Poland	Germany	Relevance for migratory game-bird management
The Hunting Law Act applies nationwide.	Federal Hunting Act (Bundesjagdgesetz) applies country-wide, supplemented by additional regulations set individually by each federal state.	Direct. Determines whether hunting rules are nationally uniform or can be regionally adjusted, which is central for season timing and management of migratory birds.
A separate regulation specifies the list of game species, classifying animals eligible for harvest.	The list of game species is embedded in the basic federal statute as “animals subject to hunting law,” indicating the taxa covered, although not all listed species may be legally harvested.	Direct. Defines which bird taxa may fall under hunting law and therefore whether they can be legally managed or harvested.
Free-living game animals are the property of the State Treasury.	Free-living game animals have no owner.	Contextual. Reflects different legal concepts of game ownership; it does not directly determine bird harvest but shapes the institutional framing of hunting rights.
Harvested game becomes the property of the manager or leaseholder of the hunting district.	Harvested game belongs to the hunter who takes it.	Contextual/indirect. May influence incentives and responsibilities after harvest, but does not directly regulate migratory bird seasons or quotas.
Every hunter is a member of the Polish Hunting Association (PZŁ)	A hunter is not required to belong to the Deutscher Jagdverband (DJV).	Contextual. Indicates the degree of organisational centralisation and potential coordination capacity, but is not itself a direct bird-management rule.
The PZŁ is the principal body supervising all hunting districts, hunting clubs, and hunting regions.	Each federal state supervises hunting management within its territory.	Direct/indirect. Relevant to governance architecture because it determines whether management oversight is centralised or regionally distributed.
Hunting districts are leased to hunting clubs that operate within the PZŁ structure.	Hunting districts are most often leased by private individuals.	Contextual/indirect. Shapes who implements hunting management locally and how responsibilities are organised, but does not directly set bird harvest rules.
A hunting district is leased from public administrative units such as the State Forests or the competent County Office.	A district is leased from private landowners.	Contextual. Describes the institutional basis of leaseholding and land access; relevant to governance context rather than directly to migratory bird regulation.
An area designated as a hunting district may not be smaller than 3000 ha.	An area may be designated a hunting district starting at 75 ha (or 150 ha for communal districts).	Indirect. Affects the spatial scale of hunting management and potential coordination across habitats used by migratory birds.
Landowners do not automatically hold hunting rights on their property.	A landowner whose parcel meets the 75 ha threshold automatically holds the hunting right.	Contextual/indirect. Influences access, local control and decentralisation of hunting rights, but does not directly determine bird harvest limits.
A hunting district is leased for a minimum term of 10 years.	A hunting district is typically leased for 9–12 years, depending on the federal state.	Contextual. Indicates continuity of management responsibility; not directly related to migratory bird harvest, but relevant to long-term governance capacity.
The right to hunt within a district rests with a club member whose club includes the district, or with a person authorised by the club board.	Hunting rights in a district rest with the owner or a person authorised by the owner (the rule of one hunter per 100 ha).	Indirect. Affects access and potential hunting pressure by defining who may hunt in a given area.
Game-damage compensation is paid by the leaseholder or manager of the district (i.e., the hunting club). The individual hunter bears no financial liability.	Game-damage compensation is paid by the district leaseholder-the hunter bears the financial liability.	Indirect/direct for conflict species. Relevant where crop damage influences tolerance and management demand, especially for geese, although standard statutory schemes often focus on mammals.
Compensation is paid for damage caused by moose, wild boar, red deer, roe deer, and fallow deer (for moose, the payment is made by the State Treasury).	Compensation covers damage caused by wild boar, red deer, roe deer, fallow deer, mouflon, chamois, wild rabbit, and pheasant.	Directly relevant as an exclusion. Shows that geese are generally outside standard statutory compensation schemes, which is important for interpreting goose-management conflicts and incentives.
Annual hunting plans are generally informed by inventories, but migratory game birds are exempt from the statutory pre-season inventory; for these species, planned take is usually based on previous harvest.	The primary factor governing the size of the harvest plan is the extent of game damage caused by the species concerned.	Direct. Relates to whether bird-harvest planning is linked to abundance estimates, previous harvest or damage-related objectives.

#### Game Species List and Legal Status in Poland and Germany (S4)

The legal amendment effective from 2 January 2026 introduced year-round protection for the hazel grouse and four other waterbird species previously listed as game in Poland. However, because this amendment falls outside the 2012/2013–2023/2024 study period, it was not included in the comparative harvest analysis. During the study period, Polish law classified 28 species as game, including 13 bird species [Regulation of 11 March 2005 - Ordinance on Establishing the List of Game Species (Journal of Laws 2005 No. 45, item 433; consolidated text Journal of Laws 2023 item 2454)]. By contrast, in Germany hazel grouse is listed as *Haselwild* under the Federal Hunting Act but enjoys a year-round closed season and is not hunted in practice.

Under the Federal Hunting Act, a broad catalogue of bird taxa is subject to hunting law, with open seasons and year-round protection rules specified by the federal states [Federal Hunting Act, Regulation on hunting seasons of 2 April 1977, as amended in 2018]. Compared with Poland, Germany’s catalogue of huntable birds is broader. For example, the Eurasian collared dove (*Streptopelia decaocto*), mute swan (*Cygnus olor*), brent goose (*Branta bernicla*), Canada goose (*Branta canadensis*), several ducks- Eurasian wigeon (*Mareca penelope*), Northern pintail (*Anas acuta*), greater scaup (*Aythya marila*), scoters (*Melanitta fusca, Melanitta nigra*) and several gulls-black-headed (*Chroicocephalus ridibundus*), common (*Larus canus*), herring (*Larus argentatus*), great black-backed (*Larus marinus*) and lesser black-backed (*Larus fuscus*)-are huntable in at least some German federal states (elsewhere closed year-round) [Regulation on hunting seasons of 2 April 1977 (Federal Law Gazette I p. 531), as last amended by Article 2 of the Ordinance of 7 March 2018 (Federal Law Gazette I p. 226)]. None of these species is classified as game in Poland. Canada goose (*Branta canadensis*) is treated as an invasive alien species (IAS) rather than as a game goose. This means that its management is framed primarily under invasive-species control provisions, rather than through ordinary game-bird harvest regulation; therefore it is not included in the Polish game-bird harvest categories analysed here.

## Discussion

This study documents persistent cross-country differences in season design and reported harvest patterns under two contrasting governance architectures. These associations are consistent with the idea that regulatory structure may shape the management opportunity space. However, the design does not identify a causal effect of governance on population outcomes or management effectiveness.

Differences in harvest totals and trends may partly reflect differences in reporting systems, compliance, and hunter effort, in addition to ecological drivers. Although we harmonised taxonomic categories to improve comparability, harvest statistics remain an imperfect proxy of actual harvest pressure because reporting incentives, enforcement intensity, and participation can vary across jurisdictions and over time. These factors could bias cross-country contrasts and should be considered when interpreting effect sizes.

Placing harvest totals in the context of population size highlights the scale of these contrasts, but the resulting ratios must be interpreted cautiously because the reference populations differ in phenological phase. Wood pigeons number 1.0–1.3 million breeding pairs in Poland and 2.9–3.5 million in Germany, yet Poland harvests about 12,000 birds a year (≈0.5-0.6% of the available national breeding estimate) while Germany harvests about 426,654 (≈ 6-11%). Woodcock estimates also refer to breeding populations (PL 20,000-100,000; DE 20,000-39,000 breeding pairs), and reported harvest corresponds to <1% of the Polish reference estimate and approximately 13–20% of the German reference estimate. However, for both wood pigeon and woodcock, autumn and winter harvest may include migrants originating from breeding areas outside the country where harvest is recorded; these ratios therefore should not be interpreted as demographic mortality imposed on local breeding populations. For geese, the reference estimates are more closely aligned with the hunting period because they refer mainly to wintering or migration-period abundance. In this group, the pattern reverses: Poland’s annual harvest of 9000-11,000 birds corresponds to ≈22-27% of the available wintering or migration-period reference population, whereas Germany’s 78,000-108,000 birds represent ≈13-18%, even though both countries are important staging and wintering areas for migratory goose populations (Fox et al. 2010; Ławicki et al. 2010; Polakowski et al. 2021). Together, these results indicate that between-country differences concern not only absolute harvest totals but also the scale of reported harvest relative to available reference populations, and that these contrasts differ by taxon. These values should be interpreted as descriptive indicators rather than direct estimates of demographic harvest mortality.

At the same time, reported harvest patterns cannot be attributed to governance alone. Differences in harvest levels may reflect (i) habitat availability and composition (especially for forest-associated species such as woodcock), (ii) hunter density, access and effort, (iii) incentives and compensation mechanisms related to crop damage, and (iv) uncertainty and comparability limits of bag statistics and population estimates. A further limitation is the lack of harmonised species-specific hunting-effort data. The number of licensed hunters or association members provides only broad institutional context and cannot be used to infer effort directed at geese, woodcock or wood pigeon. Consequently, differences in national bag size may reflect not only abundance and regulation, but also variation in hunting access, participation, reporting compliance and taxon-specific hunting preferences. For this reason, our results should not be interpreted as demonstrating that governance differences determine population status. Rather, they indicate that governance can facilitate or constrain management responses and may contribute to cross-border mismatches in harvest timing and intensity. This is particularly relevant in border regions used by the same migratory populations on short time scales-for example, geese occurring during autumn and winter in the Warta estuary in western Poland and neighbouring Brandenburg-where population-level outcomes may depend on the combined effects of multiple jurisdictions.

The contrast between the two systems should not be reduced to a simple centralised-versus-federal ranking. Greater local flexibility may improve responsiveness to phenology or crop-damage concerns, but it may also increase fragmentation and uneven implementation. Greater uniformity may enhance legal coherence and comparability but may respond less readily to local ecological variation. For shared migratory populations, the core issue is therefore not whether regulatory variation exists, but whether such variation is coordinated, evidence-based and compatible with common conservation baselines. Spatially differentiated rules may be appropriate where bird distribution, migration timing or agricultural conflicts differ among regions, but they require harmonised monitoring and transparent criteria to avoid unmanaged cumulative pressure across the flyway. Against this background, two governance-related mechanisms appear especially relevant for the game-bird taxa analysed here. First, Germany’s federal structure creates within-country heterogeneity: sixteen federal states can refine seasons and apply exemptions, producing a mosaic of management windows. Poland’s nationally fixed regulations, by contrast, favour uniformity and rapid nationwide adjustment but offer limited flexibility for local population shifts or crop-damage contexts (Mesinger and Ocieczek, 2021; Kupren and Hakuć-Błażowska, 2021). Second, crop-damage considerations can shape management incentives for herbivorous birds. Poland’s rules do not explicitly provide statutory compensation for losses from geese, despite growing evidence of such potential impacts (Polakowski et al. 2023). In Germany, statutory crop-damage compensation under the Federal Hunting Act similarly excludes geese, but several federal states have developed additional schemes addressing goose damage. In practice, such institutional arrangements may influence tolerance thresholds and the demand for preventive harvesting in areas with large wintering concentrations.

Season timing for wood pigeons and woodcocks illustrates how these governance differences may translate into biologically relevant regulatory outcomes. In Poland, wood pigeon hunting is open from 15 August to 30 November and woodcock from 1 September to 21 December, which overlaps the tail end of the breeding cycle: wood pigeons nest into October; woodcock broods typically fledge in late July-August (Toms, 2014; Brewin et al. 2022; Massimino et al. 2023; Heward et al. 2024). In Germany, federal minimum seasons start later (wood pigeon 1 November-31 January; woodcock 16 October-15 January), which tends to reduce overlap with late breeding, although state-level competence can introduce earlier openings in particular states (e.g., Brandenburg for wood pigeon) and stricter measures elsewhere (e.g., a long-standing woodcock moratorium in Hesse). Such multilevel flexibility may allow more responsive adjustments to local crop damage or population concerns, whereas a nationally uniform system may be slower to accommodate regional phenological variation. Given ongoing climate warming and documented shifts in breeding and migration phenology, periodic review of season timing-ideally at multi-year intervals-would help maintain alignment between legal windows and species’ life-history stages (Huntley et al. 2007; Devictor et al. 2008; Pearce-Higgins and Green, 2014; Lehikoinen et al. 2019).

These findings identify where more explicitly adaptive cross-border coordination may be worth testing, particularly for migratory geese in recurrent agricultural-conflict settings. A quota-based Adaptive Harvest Management (AHM) framework has been implemented since 2013/14 for the Svalbard pink-footed goose under the AEWA framework, jointly managed by Denmark and Norway, using annual spring counts, breeding success indices and the previous season’s hunting mortality to set harvest limits (Madsen et al. 2017). While such full AHM is data- and governance-intensive, it provides a useful model for linking harvest decisions to explicit objectives and updated monitoring. Rather than advocating immediate comprehensive AHM for all species, we propose a tiered adaptive framework for transboundary populations: (i) harmonised harvest reporting and shared abundance indices as a minimum requirement; (ii) predefined trigger rules (e.g., conservative reductions in seasons or quotas when indices fall below thresholds); and (iii) full AHM only where monitoring intensity and management conflict justify the investment (e.g., goose populations linked to recurrent crop damage). Any numeric harvest caps should be treated as provisional and population-specific. Robust thresholds require demographic evidence (survival, recruitment, and flyway-level abundance) and formal sustainability analyses for each focal population.

Centralised and federal governance structures appear to create different degrees of regulatory flexibility, reflected in season design and associated with differences in reported harvest-to-abundance ratios. However, we do not demonstrate population-level impacts of harvest, nor do we disentangle governance effects from ecological and social drivers. A prudent next step is to prioritise cross-border harmonisation of harvest reporting and shared abundance indicators, while reserving stronger adaptive instruments for cases where monitoring, demographic evidence, and institutional capacity are demonstrably sufficient.

## Supplementary information

Supplementary information

Supplementary information

Supplementary information

Supplementary information

## Data Availability

This study uses only publicly available data. All datasets are cited in the article and can be accessed from their original sources.
