# Supplementary material for: Regulatory Mismatch and Transboundary Governance of Migratory Game Birds: A Comparison of Poland and Germany
Source: Environ Manage. 2026 Sep 16;76(10):318. doi: 10.1007/s00267-026-02584-0 (PMC13582406; doi:10.1007/s00267-026-02584-0)
Supplement: Supplementary file 1 [file 267_2026_2584_MOESM1_ESM.docx]

For Poland, the reported national totals correspond directly to the analysed taxa–raw and harmonized values are identical (no correction applied). Harmonized values were obtained by applying a proportional adjustment for pigeons (share of “Ringeltaube” within *Wildtauben*) and a seasonal subtraction of the estimated non-native component for geese (share of *Alopochen aegyptiaca* and *Branta canadensis* within *Wildgänse*).

Tab. 1. Harmonisation of German basket harvest categories used for cross-country comparisons.

| **Taxon (reported / compared)** | **Germany: raw national basket category** | **Harmonized “common species” target (used in PL–DE comparison)** | **Rationale (why harmonization was needed)** | **Harmonization approach** | **Parameters used (central + sensitivity)** | **Empirical basis (data used to set parameters)** |
| --- | --- | --- | --- | --- | --- | --- |
| Geese (*Anser spp.*) | Wildgänse (DJV) | “*Anser* (common species)” (approx. native *Anser*; excluding non-native geese) | German *Wildgänse* totals include additional non-native species harvested in Germany but not included in the Polish reference category (raw totals are not taxonomically equivalent). | Annual reduction of the raw basket total by the estimated seasonal share of non-native geese (Egyptian goose *Alopochen aegyptiaca*, Canada goose *Branta canadensis*). | Seasonal share of non-native geese qt (time-varying). Observed range in this dataset: ~12.8% (2012/13) to ~39.0% (2023/24). | Länder-level reports providing species breakdowns of goose harvest (e.g., NRW; Lower Saxony) + DJV national totals (literature confirming establishment/spread of non-native geese). |
| Common wood pigeon (*Columba palumbus*) | Wildtauben (DJV) | “*C. palumbus* (common species)” | National German pigeon harvest is reported only as an aggregate basket. Species-level split is not mandatory at the federal level. | Proportional conversion of *Wildtauben* to wood pigeon using the share of “Ringeltaube” within the basket. | Wood pigeon share p (assumed constant): p = 0.995 (central). Sensitivity: p = 0.992; 0.9985; 0.9993 (based on regional variation). | Länder-level reports where *Wildtauben* is disaggregated into “Ringeltaube” vs other categories (e.g., *Streptopelia decaocto*, “übrige Wildtauben”) + DJV national totals. |

Tab. 2. Raw and harmonized annual harvest totals for geese, Eurasian woodcock and common wood pigeon in Poland and Germany, 2012/2013–2023/2024.

| **Season** | **Country** | **Taxon** | **Series type** | **Harvest** | **Basket definition** | **Notes** |
| --- | --- | --- | --- | --- | --- | --- |
| **2012/2013** | PL | Geese (*Anser spp.*) | raw_basket | 9600 | PL official category | Raw basket = harmonized for PL |
| **2012/2013** | PL | Geese (*Anser spp.*) | harmonized_common | 9600 | PL official category | Raw basket = harmonized for PL |
| **2012/2013** | DE | Geese (*Anser spp.*) | raw_basket | 78296 | DE Wildgänse basket | Raw basket total |
| **2012/2013** | DE | Geese (*Anser spp.*) | harmonized_common | 68296 | DE Wildgänse basket | Harmonized common-species series (approx. *Anser*) |
| **2012/2013** | PL | Eurasian woodcock (*Scolopax rusticola*) | reported | 400 | Species-level reported | No harmonization applied |
| **2012/2013** | DE | Eurasian woodcock (*Scolopax rusticola*) | reported | 11811 | Species-level reported | No harmonization applied |
| **2012/2013** | PL | Common wood pigeon (*Columba palumbus*) | raw_basket | 13500 | PL official category | Raw basket = harmonized for PL |
| **2012/2013** | PL | Common wood pigeon (*Columba palumbus*) | harmonized_common | 13500 | PL official category | Raw basket = harmonized for PL |
| **2012/2013** | DE | Common wood pigeon (*Columba palumbus*) | raw_basket | 704151 | DE Wildtauben basket | Raw basket total |
| **2012/2013** | DE | Common wood pigeon (*Columba palumbus*) | harmonized_common | 700630 | DE Wildtauben basket | Harmonized common-species series (approx. *C. palumbus*) |
| **2013/2014** | PL | Geese (*Anser spp.*) | raw_basket | 10900 | PL official category | Raw basket = harmonized for PL |
| **2013/2014** | PL | Geese (*Anser spp.*) | harmonized_common | 10900 | PL official category | Raw basket = harmonized for PL |
| **2013/2014** | DE | Geese (*Anser spp.*) | raw_basket | 81754 | DE Wildgänse basket | Raw basket total |
| **2013/2014** | DE | Geese (*Anser spp.*) | harmonized_common | 69254 | DE Wildgänse basket | Harmonized common-species series (approx. *Anser*) |
| **2013/2014** | PL | Eurasian woodcock (*Scolopax rusticola*) | reported | 400 | Species-level reported | No harmonization applied |
| **2013/2014** | DE | Eurasian woodcock (*Scolopax rusticola*) | reported | 10954 | Species-level reported | No harmonization applied |
| **2013/2014** | PL | Common wood pigeon (*Columba palumbus*) | raw_basket | 11800 | PL official category | Raw basket = harmonized for PL |
| **2013/2014** | PL | Common wood pigeon (*Columba palumbus*) | harmonized_common | 11800 | PL official category | Raw basket = harmonized for PL |
| **2013/2014** | DE | Common wood pigeon (*Columba palumbus*) | raw_basket | 578735 | DE Wildtauben basket | Raw basket total |
| **2013/2014** | DE | Common wood pigeon (*Columba palumbus*) | harmonized_common | 575841 | DE Wildtauben basket | Harmonized common-species series (approx. *C. palumbus*) |
| **2014/2015** | PL | Geese (*Anser spp.*) | raw_basket | 10300 | PL official category | Raw basket = harmonized for PL |
| **2014/2015** | PL | Geese (*Anser spp.*) | harmonized_common | 10300 | PL official category | Raw basket = harmonized for PL |
| **2014/2015** | DE | Geese (*Anser spp.*) | raw_basket | 83059 | DE Wildgänse basket | Raw basket total |
| **2014/2015** | DE | Geese (*Anser spp.*) | harmonized_common | 67059 | DE Wildgänse basket | Harmonized common-species series (approx. *Anser*) |
| **2014/2015** | PL | Eurasian woodcock (*Scolopax rusticola*) | reported | 500 | Species-level reported | No harmonization applied |
| **2014/2015** | DE | Eurasian woodcock (*Scolopax rusticola*) | reported | 9672 | Species-level reported | No harmonization applied |
| **2014/2015** | PL | Common wood pigeon (*Columba palumbus*) | raw_basket | 12100 | PL official category | Raw basket = harmonized for PL |
| **2014/2015** | PL | Common wood pigeon (*Columba palumbus*) | harmonized_common | 12100 | PL official category | Raw basket = harmonized for PL |
| **2014/2015** | DE | Common wood pigeon (*Columba palumbus*) | raw_basket | 552340 | DE Wildtauben basket | Raw basket total |
| **2014/2015** | DE | Common wood pigeon (*Columba palumbus*) | harmonized_common | 549578 | DE Wildtauben basket | Harmonized common-species series (approx. *C. palumbus*) |
| **2015/2016** | PL | Geese (*Anser spp.*) | raw_basket | 9600 | PL official category | Raw basket = harmonized for PL |
| **2015/2016** | PL | Geese (*Anser spp.*) | harmonized_common | 9600 | PL official category | Raw basket = harmonized for PL |
| **2015/2016** | DE | Geese (*Anser spp.*) | raw_basket | 96217 | DE Wildgänse basket | Raw basket total |
| **2015/2016** | DE | Geese (*Anser spp.*) | harmonized_common | 75217 | DE Wildgänse basket | Harmonized common-species series (approx. *Anser*) |
| **2015/2016** | PL | Eurasian woodcock (*Scolopax rusticola*) | reported | 400 | Species-level reported | No harmonization applied |
| **2015/2016** | DE | Eurasian woodcock (*Scolopax rusticola*) | reported | 8835 | Species-level reported | No harmonization applied |
| **2015/2016** | PL | Common wood pigeon (*Columba palumbus*) | raw_basket | 12400 | PL official category | Raw basket = harmonized for PL |
| **2015/2016** | PL | Common wood pigeon (*Columba palumbus*) | harmonized_common | 12400 | PL official category | Raw basket = harmonized for PL |
| **2015/2016** | DE | Common wood pigeon (*Columba palumbus*) | raw_basket | 509706 | DE Wildtauben basket | Raw basket total |
| **2015/2016** | DE | Common wood pigeon (*Columba palumbus*) | harmonized_common | 507157 | DE Wildtauben basket | Harmonized common-species series (approx. *C. palumbus*) |
| **2016/2017** | PL | Geese (*Anser spp.*) | raw_basket | 9100 | PL official category | Raw basket = harmonized for PL |
| **2016/2017** | PL | Geese (*Anser spp.*) | harmonized_common | 9100 | PL official category | Raw basket = harmonized for PL |
| **2016/2017** | DE | Geese (*Anser spp.*) | raw_basket | 94331 | DE Wildgänse basket | Raw basket total |
| **2016/2017** | DE | Geese (*Anser spp.*) | harmonized_common | 69331 | DE Wildgänse basket | Harmonized common-species series (approx. *Anser*) |
| **2016/2017** | PL | Eurasian woodcock (*Scolopax rusticola*) | reported | 400 | Species-level reported | No harmonization applied |
| **2016/2017** | DE | Eurasian woodcock (*Scolopax rusticola*) | reported | 7829 | Species-level reported | No harmonization applied |
| **2016/2017** | PL | Common wood pigeon (*Columba palumbus*) | raw_basket | 12100 | PL official category | Raw basket = harmonized for PL |
| **2016/2017** | PL | Common wood pigeon (*Columba palumbus*) | harmonized_common | 12100 | PL official category | Raw basket = harmonized for PL |
| **2016/2017** | DE | Common wood pigeon (*Columba palumbus*) | raw_basket | 453913 | DE Wildtauben basket | Raw basket total |
| **2016/2017** | DE | Common wood pigeon (*Columba palumbus*) | harmonized_common | 451643 | DE Wildtauben basket | Harmonized common-species series (approx. *C. palumbus*) |
| **2017/2018** | PL | Geese (*Anser spp.*) | raw_basket | 10500 | PL official category | Raw basket = harmonized for PL |
| **2017/2018** | PL | Geese (*Anser spp.*) | harmonized_common | 10500 | PL official category | Raw basket = harmonized for PL |
| **2017/2018** | DE | Geese (*Anser spp.*) | raw_basket | 95394 | DE Wildgänse basket | Raw basket total |
| **2017/2018** | DE | Geese (*Anser spp.*) | harmonized_common | 66394 | DE Wildgänse basket | Harmonized common-species series (approx. *Anser*) |
| **2017/2018** | PL | Eurasian woodcock (*Scolopax rusticola*) | reported | 450 | Species-level reported | No harmonization applied |
| **2017/2018** | DE | Eurasian woodcock (*Scolopax rusticola*) | reported | 8570 | Species-level reported | No harmonization applied |
| **2017/2018** | PL | Common wood pigeon (*Columba palumbus*) | raw_basket | 11500 | PL official category | Raw basket = harmonized for PL |
| **2017/2018** | PL | Common wood pigeon (*Columba palumbus*) | harmonized_common | 11500 | PL official category | Raw basket = harmonized for PL |
| **2017/2018** | DE | Common wood pigeon (*Columba palumbus*) | raw_basket | 431047 | DE Wildtauben basket | Raw basket total |
| **2017/2018** | DE | Common wood pigeon (*Columba palumbus*) | harmonized_common | 428892 | DE Wildtauben basket | Harmonized common-species series (approx. *C. palumbus*) |
| **2018/2019** | PL | Geese (*Anser spp.*) | raw_basket | 11000 | PL official category | Raw basket = harmonized for PL |
| **2018/2019** | PL | Geese (*Anser spp.*) | harmonized_common | 11000 | PL official category | Raw basket = harmonized for PL |
| **2018/2019** | DE | Geese (*Anser spp.*) | raw_basket | 107604 | DE Wildgänse basket | Raw basket total |
| **2018/2019** | DE | Geese (*Anser spp.*) | harmonized_common | 72604 | DE Wildgänse basket | Harmonized common-species series (approx. *Anser*) |
| **2018/2019** | PL | Eurasian woodcock (*Scolopax rusticola*) | reported | 490 | Species-level reported | No harmonization applied |
| **2018/2019** | DE | Eurasian woodcock (*Scolopax rusticola*) | reported | 8442 | Species-level reported | No harmonization applied |
| **2018/2019** | PL | Common wood pigeon (*Columba palumbus*) | raw_basket | 11900 | PL official category | Raw basket = harmonized for PL |
| **2018/2019** | PL | Common wood pigeon (*Columba palumbus*) | harmonized_common | 11900 | PL official category | Raw basket = harmonized for PL |
| **2018/2019** | DE | Common wood pigeon (*Columba palumbus*) | raw_basket | 403567 | DE Wildtauben basket | Raw basket total |
| **2018/2019** | DE | Common wood pigeon (*Columba palumbus*) | harmonized_common | 401549 | DE Wildtauben basket | Harmonized common-species series (approx. *C. palumbus*) |
| **2019/2020** | PL | Geese (*Anser spp.*) | raw_basket | 10200 | PL official category | Raw basket = harmonized for PL |
| **2019/2020** | PL | Geese (*Anser spp.*) | harmonized_common | 10200 | PL official category | Raw basket = harmonized for PL |
| **2019/2020** | DE | Geese (*Anser spp.*) | raw_basket | 112099 | DE Wildgänse basket | Raw basket total |
| **2019/2020** | DE | Geese (*Anser spp.*) | harmonized_common | 72099 | DE Wildgänse basket | Harmonized common-species series (approx. *Anser*) |
| **2019/2020** | PL | Eurasian woodcock (*Scolopax rusticola*) | reported | 450 | Species-level reported | No harmonization applied |
| **2019/2020** | DE | Eurasian woodcock (*Scolopax rusticola*) | reported | 9983 | Species-level reported | No harmonization applied |
| **2019/2020** | PL | Common wood pigeon (*Columba palumbus*) | raw_basket | 11500 | PL official category | Raw basket = harmonized for PL |
| **2019/2020** | PL | Common wood pigeon (*Columba palumbus*) | harmonized_common | 11500 | PL official category | Raw basket = harmonized for PL |
| **2019/2020** | DE | Common wood pigeon (*Columba palumbus*) | raw_basket | 369549 | DE Wildtauben basket | Raw basket total |
| **2019/2020** | DE | Common wood pigeon (*Columba palumbus*) | harmonized_common | 367701 | DE Wildtauben basket | Harmonized common-species series (approx. *C. palumbus*) |
| **2020/2021** | PL | Geese (*Anser spp.*) | raw_basket | 11900 | PL official category | Raw basket = harmonized for PL |
| **2020/2021** | PL | Geese (*Anser spp.*) | harmonized_common | 11900 | PL official category | Raw basket = harmonized for PL |
| **2020/2021** | DE | Geese (*Anser spp.*) | raw_basket | 115424 | DE Wildgänse basket | Raw basket total |
| **2020/2021** | DE | Geese (*Anser spp.*) | harmonized_common | 71424 | DE Wildgänse basket | Harmonized common-species series (approx. *Anser*) |
| **2020/2021** | PL | Eurasian woodcock (*Scolopax rusticola*) | reported | 300 | Species-level reported | No harmonization applied |
| **2020/2021** | DE | Eurasian woodcock (*Scolopax rusticola*) | reported | 4197 | Species-level reported | No harmonization applied |
| **2020/2021** | PL | Common wood pigeon (*Columba palumbus*) | raw_basket | 10500 | PL official category | Raw basket = harmonized for PL |
| **2020/2021** | PL | Common wood pigeon (*Columba palumbus*) | harmonized_common | 10500 | PL official category | Raw basket = harmonized for PL |
| **2020/2021** | DE | Common wood pigeon (*Columba palumbus*) | raw_basket | 303021 | DE Wildtauben basket | Raw basket total |
| **2020/2021** | DE | Common wood pigeon (*Columba palumbus*) | harmonized_common | 301506 | DE Wildtauben basket | Harmonized common-species series (approx. *C. palumbus*) |
| **2021/2022** | PL | Geese (*Anser spp.*) | raw_basket | 13300 | PL official category | Raw basket = harmonized for PL |
| **2021/2022** | PL | Geese (*Anser spp.*) | harmonized_common | 13300 | PL official category | Raw basket = harmonized for PL |
| **2021/2022** | DE | Geese (*Anser spp.*) | raw_basket | 115332 | DE Wildgänse basket | Raw basket total |
| **2021/2022** | DE | Geese (*Anser spp.*) | harmonized_common | 69332 | DE Wildgänse basket | Harmonized common-species series (approx. *Anser*) |
| **2021/2022** | PL | Eurasian woodcock (*Scolopax rusticola*) | reported | 240 | Species-level reported | No harmonization applied |
| **2021/2022** | DE | Eurasian woodcock (*Scolopax rusticola*) | reported | 10034 | Species-level reported | No harmonization applied |
| **2021/2022** | PL | Common wood pigeon (*Columba palumbus*) | raw_basket | 9600 | PL official category | Raw basket = harmonized for PL |
| **2021/2022** | PL | Common wood pigeon (*Columba palumbus*) | harmonized_common | 9600 | PL official category | Raw basket = harmonized for PL |
| **2021/2022** | DE | Common wood pigeon (*Columba palumbus*) | raw_basket | 300002 | DE Wildtauben basket | Raw basket total |
| **2021/2022** | DE | Common wood pigeon (*Columba palumbus*) | harmonized_common | 298502 | DE Wildtauben basket | Harmonized common-species series (approx. *C. palumbus*) |
| **2022/2023** | PL | Geese (*Anser spp.*) | raw_basket | 12800 | PL official category | Raw basket = harmonized for PL |
| **2022/2023** | PL | Geese (*Anser spp.*) | harmonized_common | 12800 | PL official category | Raw basket = harmonized for PL |
| **2022/2023** | DE | Geese (*Anser spp.*) | raw_basket | 141471 | DE Wildgänse basket | Raw basket total |
| **2022/2023** | DE | Geese (*Anser spp.*) | harmonized_common | 89471 | DE Wildgänse basket | Harmonized common-species series (approx. *Anser*) |
| **2022/2023** | PL | Eurasian woodcock (*Scolopax rusticola*) | reported | 200 | Species-level reported | No harmonization applied |
| **2022/2023** | DE | Eurasian woodcock (*Scolopax rusticola*) | reported | 12149 | Species-level reported | No harmonization applied |
| **2022/2023** | PL | Common wood pigeon (*Columba palumbus*) | raw_basket | 8300 | PL official category | Raw basket = harmonized for PL |
| **2022/2023** | PL | Common wood pigeon (*Columba palumbus*) | harmonized_common | 8300 | PL official category | Raw basket = harmonized for PL |
| **2022/2023** | DE | Common wood pigeon (*Columba palumbus*) | raw_basket | 275130 | DE Wildtauben basket | Raw basket total |
| **2022/2023** | DE | Common wood pigeon (*Columba palumbus*) | harmonized_common | 273754 | DE Wildtauben basket | Harmonized common-species series (approx. *C. palumbus*) |
| **2023/2024** | PL | Geese (*Anser spp.*) | raw_basket | 13200 | PL official category | Raw basket = harmonized for PL |
| **2023/2024** | PL | Geese (*Anser spp.*) | harmonized_common | 13200 | PL official category | Raw basket = harmonized for PL |
| **2023/2024** | DE | Geese (*Anser spp.*) | raw_basket | 148851 | DE Wildgänse basket | Raw basket total |
| **2023/2024** | DE | Geese (*Anser spp.*) | harmonized_common | 90851 | DE Wildgänse basket | Harmonized common-species series (approx. *Anser*) |
| **2023/2024** | PL | Eurasian woodcock (*Scolopax rusticola*) | reported | 170 | Species-level reported | No harmonization applied |
| **2023/2024** | DE | Eurasian woodcock (*Scolopax rusticola*) | reported | 14424 | Species-level reported | No harmonization applied |
| **2023/2024** | PL | Common wood pigeon (*Columba palumbus*) | raw_basket | 8200 | PL official category | Raw basket = harmonized for PL |
| **2023/2024** | PL | Common wood pigeon (*Columba palumbus*) | harmonized_common | 8200 | PL official category | Raw basket = harmonized for PL |
| **2023/2024** | DE | Common wood pigeon (*Columba palumbus*) | raw_basket | 264417 | DE Wildtauben basket | Raw basket total |
| **2023/2024** | DE | Common wood pigeon (*Columba palumbus*) | harmonized_common | 263095 | DE Wildtauben basket | Harmonized common-species series (approx. *C. palumbus*) |
