# Supplementary material for: Regulatory Mismatch and Transboundary Governance of Migratory Game Birds: A Comparison of Poland and Germany
Source: Environ Manage. 2026 Sep 16;76(10):318. doi: 10.1007/s00267-026-02584-0 (PMC13582406; doi:10.1007/s00267-026-02584-0)
Supplement: Supplementary file 2 [file 267_2026_2584_MOESM2_ESM.docx]

1. Act of 13 October 1995 - Hunting Law (Journal of Laws of 2023, item 1082)
2. Regulation of the Minister of the Environment of 11 March 2005 on establishing the list of game‑animal species (Journal of Laws 2023, item 2454) – establishes the list of species recognised as game.
3. Regulation of the Minister of the Environment of 16 March 2005 on determining hunting seasons for game animals (Journal of Laws 2023, item 2455) – sets the periods during which individual game species may be hunted.
4. Regulation of the Minister of the Environment of 23 March 2005 on detailed conditions for hunting and marking carcasses (Journal of Laws 2005, No. 61, item 548, as amended) – specifies rules for conducting hunts and marking harvested game.
5. Regulation of the Minister of the Environment of 22 September 2009 on hunting licences (Journal of Laws 2009, No. 174, item 1356, as amended) – lays down the requirements and procedures for obtaining the right to hunt.
6. Regulation of the Minister of the Environment of 28 December 2009 on sanitary‑veterinary conditions for the harvesting of wild animals (Journal of Laws 2010, No. 6, item 38, as amended) – sets hygiene and veterinary‑control rules when taking wild animals.
7. Regulation of the Minister of the Environment of 29 September 2010 on annual hunting plans (Journal of Laws 2010, No. 186, item 1249, as amended) – governs preparation and implementation of yearly hunting plans.
8. Regulation of the Minister of the Environment of 20 March 2012 on detailed conditions for sanitary culling of game animals (Journal of Laws 2012, item 327, as amended) – defines procedures and conditions for sanitary culls.
9. Regulation of the Minister of the Environment of 8 March 2018 on the assessment of hunting damage (Journal of Laws 2018, item 827, as amended) – regulates evaluation and compensation of damage caused by game.
10. Regulation of the Minister of the Environment of 16 April 2019 on criteria for assessing hunting trophies (Journal of Laws 2019, item 759, as amended) – sets standards for evaluating trophies taken during hunts.
11. Federal Hunting Act (Bundesjagdgesetz) of 29 November 1952 (Federal Law Gazette I p. 837, as amended).
12. Regulation on hunting seasons of 2 April 1977 (Federal Law Gazette I p. 531)
13. Weapons Act (Waffengesetz) of 11 October 2002 (Federal Law Gazette I p. 3970) – rules on acquisition and use of firearms by hunters.
14. Animal Welfare Act (Tierschutzgesetz) of 24 July 1972 – animal‑welfare provisions relevant to hunting.
15. Federal Nature Conservation Act (Bundesnaturschutzgesetz) of 29 July 2009 – habitat‑protection and sustainable‑hunting provisions.
16. German state (Land) hunting acts:

- Hunting Act of Baden‑Württemberg (Landesjagdgesetz BW)
- Bavarian Hunting Act (Bayerisches Jagdgesetz)
- Berlin Hunting Act (Berliner Jagdgesetz)
- Brandenburg Hunting Act (Brandenburgisches Jagdgesetz)
- Bremen Hunting Act (Bremisches Jagdgesetz)
- Hamburg Hunting Act (Hamburgisches Jagdgesetz)
- Hesse Hunting Act (Hessisches Jagdgesetz)
- Mecklenburg‑Western Pomerania Hunting Act (Landesjagdgesetz M‑V)
- Lower Saxony Hunting Act (Niedersächsisches Jagdgesetz)
- North Rhine‑Westphalia Hunting Act (Landesjagdgesetz NRW)
- Rhineland‑Palatinate Hunting Act (Landesjagdgesetz RLP)
- Saarland Hunting Act (Saarländisches Jagdgesetz)
- Saxony Hunting Act (Sächsisches Jagdgesetz)
- Saxony‑Anhalt Hunting Act (Landesjagdgesetz LSA)
- Schleswig‑Holstein Hunting Act (Landesjagdgesetz SH)
