# Supplementary material for: Regulatory Mismatch and Transboundary Governance of Migratory Game Birds: A Comparison of Poland and Germany
Source: Environ Manage. 2026 Sep 16;76(10):318. doi: 10.1007/s00267-026-02584-0 (PMC13582406; doi:10.1007/s00267-026-02584-0)
Supplement: Supplementary file 3 [file 267_2026_2584_MOESM3_ESM.docx]

|  | Poland | | | Germany | | |  |
| --- | --- | --- | --- | --- | --- | --- | --- |
| Species | N breeding pairs (Chodkiewicz et al., 2019) | National population trend (Chylarecki et al., 2018) | Hunting season | N breeding pairs (Gerlach et al., 2019) | National population trend (Gerlach et al., 2019) | Hunting season | Global population trend (BirdLife International, 2025) |
| Common wood pigeon (*Columba palubmus*) | 1,000,000-1,300,000 | increasing **↑** | 15 VIII-30 XI | 2,900,000- 3,500,000 | increasing **↑** | 01 XI-20 II (Branderburg 1 IX-31 I) | increasing **↑** |
| Eurasian woodcock (*Scolopax rusticola*) | 20,000-100,000 | stable **−** | 1 IX-21 XII | 20,000-39,000 | decreasing (Bokämper et al., 2024) | 16 X-15 I (Hesse moratorium since 1998) | stable **−** |
| Greylag goose (*Anser anser*) | 6,000-9,000 | increasing **↑** | 1 IX-21 XII (15 I) * | 42,000-59,000 | increasing **↑** | 01 XI-15 I  (01 VIII-31 VIII, 1 IX-31 X and 16 I-31 I)** | increasing **↑** |
|  | 5,000-9,000 wintering individuals (Chodkiewicz et al., 2019) | | | 160,000 wintering individuals (Gerlach et al., 2019) | | | |
|  | 92,000 migrant individuals (IWC) (Wetlands International, 2024) | | | 244,000 migrant individuals (IWC) (Wetlands International, 2024) | | | |
| Greater white-fronted goose (*Anser albifrons*) | 0 | increasing **↑** | 1 IX-21 XII (15 I) | 0-1 | increasing **↑** | 01 XI-15 I | unknow **?** |
|  | 6,000-33,000 wintering individuals (Chodkiewicz et al., 2019) | | | 420,000 wintering individuals (Gerlach et al., 2019) | | | |
|  | 120,000-130,000 migrant individuals (IWC) (Wetlands International, 2024) | | | 300,000-320,000 migrant individuals (IWC) (Wetlands International, 2024) | | | |
| Bean goose (*Anser fabalis*) | 0 | unknow **?** | 1 IX-21 XII (15 I) | 0 | decreasing **↓** | 01 XI-15 I | decreasing **↓** |
|  | 7,000-23,000 wintering individuals (Chodkiewicz et al., 2019) | | | 11,500 wintering individuals (Gerlach et al., 2019) | | | |

* The greylag-goose season in Poland generally runs 1 IX - 21 XII, but in four western voivodeships-West Pomeranian, Lubusz, Greater Poland, and Lower Silesian-Regulation of the Ministry of Climate and Environment (Journal of Laws 2023, item 2556) extends it to 15 I. More than 70% of the national wintering stock (≈ 35-50 000 birds in mid-January) stays in these regions, and feeding flocks inflict the greatest damage on winter cereals and permanent grassland (Wetlands International 2024). Phenological analysis shows that departure for the breeding grounds begins only in late II, so a season ending 15 I complies with the Birds Directive 2009/147/EC requirement that hunting must not overlap the reproductive period or spring migration. The extension is therefore interpreted as a derogation to reduce agricultural damage rather than an expansion of recreational hunting.

** Hunting periods for the greylag goose in Germany arise from a three-tier regulatory scheme. The Birds Directive 2009/147/EC requires a total ban on shooting during the breeding season and spring migration but permits derogations to prevent “serious damage to agriculture” (European Parliament and Council, 2009). On that basis the federal Jagdzeit-V sets a maximum season of 1 VIII - 31 VIII and 1 XI - 15 I (Bundesministerium der Justiz, 1977). Each Land may shorten these dates or-if genuine losses are demonstrated-extend them solely as *Schadensabwehr* (§ 22 BJagdG). Thus Brandenburg allows shooting 1 IX - 31 X and 16 I - 31 I but only on fields with documented damage. Sachsen-Anhalt authorises hunting 1 IX - 31 X when ≥ 50 geese are feeding on the crop Schleswig-Holstein moves the season’s end to 31 I yet prohibits harvest within Natura 2000 sites. Länder with small wintering flocks and marginal damage, such as Berlin or Hesse, retain the federal calendar. The differences therefore stem from the interplay between the EU requirement to protect breeding phenology and local agricultural pressure: the greater the concentration of wintering geese and crop losses, the longer the shooting “windows”, which are formally treated as preventive measures rather than recreational hunting.

**References**

Bundesministerium der Justiz (1977) Verordnung über die Jagdzeiten (JagdzeitV) vom 2. April 1977 (BGBl. I S. 531), zuletzt geändert durch Art. 2 der Verordnung vom 7. März 2018 (BGBl. I S. 226).

Chodkiewicz T, Chylarecki P, Sikora A, Wardecki Ł, Bobrek R, Neubauer G, Kuczyński L (2019) Raport z wdrażania art. 12 Dyrektywy Ptasiej w Polsce w latach 2013–2018: stan, zmiany, zagrożenia [Article 12 Birds Directive implementation report for Poland 2013–2018: status, trends, threats]. Biul Monit Przyr 20:1–80.

Chylarecki P, Chodkiewicz T, Neubauer G, Sikora A, Meissner W, Woźniak B, Wylegała P, Ławicki Ł, Marchowski D, Betleja J, Bzoma S, Cenian Z, Górski A, Korniluk M, Moczarska J, Ochocińska D, Rubacha S, Wieloch M, Zielińska M, Zieliński P, Kuczyński L (2018) Trendy liczebności ptaków w Polsce [Bird population trends in Poland]. GIOŚ, Warsaw.

European Parliament and Council of the European Union (2009) Directive 2009/147/EC of the European Parliament and of the Council of 30 November 2009 on the conservation of wild birds. Official Journal of the European Union L 20.

Gerlach B, Dröschmeister R, Langgemach T, Borkenhagen K, Busch M, Hauswirth M, Heinicke T, Kamp J, Karthäuser J, König C, Markones N, Prior N, Trautmann S, Wahl J, Sudfeldt C (2019) Vögel in Deutschland – Übersichten zur Bestandssituation. Dachverband Deutscher Avifaunisten, Bundesamt für Naturschutz and Länderarbeitsgemeinschaft der Vogelschutzwarten, Münster.

Wetlands International (2024) International Waterbird Census online database: January 2024 country totals – Greylag Goose and Greater White-fronted Goose, Poland and Germany [Data set]. Wetlands International. Available at: <https://iwc.wetlands.org>. Accessed 20 Mar 2025.
