# Supplementary material for: Regulatory Mismatch and Transboundary Governance of Migratory Game Birds: A Comparison of Poland and Germany
Source: Environ Manage. 2026 Sep 16;76(10):318. doi: 10.1007/s00267-026-02584-0 (PMC13582406; doi:10.1007/s00267-026-02584-0)
Supplement: Supplementary file 4 [file 267_2026_2584_MOESM4_ESM.docx]

|  | Poland (species) | Germany (species) | Differences |
| --- | --- | --- | --- |
| Birds | common pheasant (*Phasianus colchicus*), grey partridge (*Perdix perdix*), hazel grouse (*Tetrastes bonasia*), common wood pigeon (*Columba palumbus*), greylag goose (*Anser anser*), bean goose (*Anser fabalis*), greater white-fronted goose (*Anser albifrons*), mallard (*Anas platyrhynchos*), Eurasian teal (*Anas crecca*), common pochard (*Aythya ferina*), tufted duck (*Aythya fuligula*), Eurasian woodcock (*Scolopax rusticola*), Eurasian coot (*Fulica atra*) | Grey partridge (*Perdix perdix*), common pheasant (*Phasianus colchicus*), wild turkey (*Meleagris gallopavo*), common wood pigeon (*Columba palumbus*), eurasian collared dove (*Streptopelia decaocto*), mute swan (*Cygnus olor*), greylag goose (*Anser anser*), greater white-fronted goose (*Anser albifrons*), bean goose (*Anser fabalis*), brent goose (*Branta bernicla*), canada goose (*Branta canadensis*), mallard (*Anas platyrhynchos*), eurasian wigeon (*Mareca penelope*), eurasian teal (Anas crecca), northern pintail (*Anas acuta*), greater scaup (*Aythya marila*), tufted duck (*Aythya fuligula*), common pochard (Aythya ferina), velvet scoter (*Melanitta fusca*), common scoter (*Melanitta nigra*), eurasian woodcock (*Scolopax rusticola*), eurasian coot (*Fulica atra*), black-headed gull (*Chroicocephalus ridibundus*), common gull (*Larus canus*), herring gull (*Larus argentatus*), great black-backed gull (*Larus marinus*), lesser black-backed gull (*Larus fuscus*) | PL: hazel grouse (*Tetrastes bonasia*)*  DE: wild turkey (*Meleagris gallopavo*), Eurasian collared dove (*Streptopelia decaocto*), mute swan (*Cygnus olor*), brent goose (*Branta bernicla*), Canada goose (*Branta canadensis*)**, Eurasian wigeon (*Mareca penelope*), northern pintail (*Anas acuta*), greater scaup (*Aythya marila*), velvet scoter (*Melanitta fusca*), common scoter (*Melanitta nigra*), black-headed gull (*Chroicocephalus ridibundus*), common gull (*Larus canus*), herring gull (*Larus argentatus*), great black-backed gull *(Larus marinus*), lesser black-backed gull (*Larus fuscus*) |

The Polish game-bird list presented here reflects the legal situation during the study period. As of 2 January 2026, five bird species-tufted duck *Aythya fuligula,* common pochard *Aythya ferina*, Eurasian coot *Fulica atra*, hazel grouse *Tetrastes bonasia* and Eurasian woodcock *Scolopax rusticola*-have been removed from the national list of game species (Regulation of the Minister of Climate and Environment, 2025)

* In Germany, has been under a year-round closed season (year-round protection)

** In Poland, they are considered IAS (invasive alien species). They are subject to shooting but formally they are not game animals.

**References**

Regulation of the Minister of Climate and Environment of 22 September 2025 on the list of game species. Journal of Laws of the Republic of Poland, 2025, item 1286.
